# Honoring Leslie A. Geddes - Farewell ...

**DOI:** 10.1186/1475-925X-9-1

**Published:** 2010-01-05

**Authors:** Max E Valentinuzzi

**Affiliations:** 1 Instituto de Ingeniería Biomédica, Facultad de Ingeniería, Universidad de Buenos Aires, Buenos Aires, Argentina

## Abstract

*Honor thy father and thy mother, say the Holy Scriptures*[1], *for they at least gave thee this biological life, but honor thy teachers, too, for they gave thee knowledge and example.*

Leslie Alexander Geddes took off on a long, long trip, Sunday October 25, 2009, leaving his body for medical and research use. The departing station was West Lafayette, Indiana, where he set foot in 1974, at Purdue University, stamping there a unique deep imprint, similar and probably more profound than the one left at Baylor College of Medicine (BCM), Houston, Texas, in the period 1955-1974. Memories came back as a flood the minute after a message broke the news to me: When I first met him visiting the Department of Physiology at BCM back in 1962, my first *Classical Physiology with Modern Instrumentation Summer Course *... The versatile Physiograph was the main equipment, an electronic-mechanical three or four channel recorder that could pick up a variety of physiological variables. Les and his collaborators had introduced also the impedance pneumograph, which was a simplified version of previous developments made by others. It became a ubiquitous unit that trod many roads in the hands of eager and curious students. Ventricular fibrillation and especially its counterpart, defibrillation, stand out as subjects occupying his concern along the years. Many were the students recruited to such effort and long is the list of papers on the subject. Physiological signals attracted considerable part of his activities because one of his perennial mottos was *measurement is essential in physiology*. He has written thirteen books and over eight hundred scientific papers, receiving also several prizes and distinctions. Not only his interests stayed within the academic environment but an industrial hue was manifested in over 20 USA patents, all applied to medical use. History of science and technology was another area in which, often with Hebbel Hoff, he uncovered astounding and delightful information. It is beyond my capability to review everything Les did, least of all what he did during the long span at Purdue.

## 

Someone once said almost with a smile and a soft grin, *not dead, just gone before *... and that is what all this is about: Forced by an unfriendly companion named pneumonia, Leslie Alexander Geddes took off on a long, long trip, Sunday October 25, 2009, not surprisingly and consistent with his generous philosophy and standings, leaving his body for medical and research use, and very likely looking for another good lab, which the Great Engineer will certainly find for him (Figure 1). The departing station was West Lafayette, Indiana, where he set foot in 1974, at Purdue University, stamping there a unique deep imprint, similar and probably more profound than the one left at Baylor College of Medicine (BCM), Houston, Texas, in the period 1955-1974.

**Figure 1:**
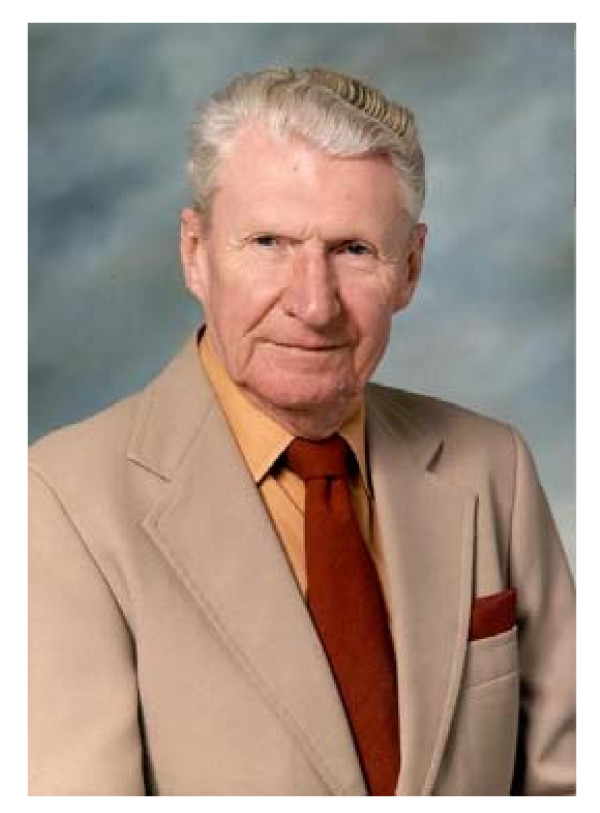
**L.A. Geddes**. May 24, 1921, Port Gordon, Scotland - Oct 25, 2009, West Lafayette, IN, USA. This picture is freely available in the WEB.

Memories came back as a flood the minute after a message broke the news to me: When I first met him visiting the Department of Physiology (BCM) a sunny Saturday morning of March 1962, a month later starting there, within a NASA Project, making respiratory measurements with the impedance technique as Lee E. Baker's assistant and under Les' supervision; Lee was then a doctoral graduate student. The just started manned space flights used that simple method to monitor the astronauts' breathing movements. And my first *Classical Physiology with Modern Instrumentation Summer Course *came as refreshing uplift that very June 1962 (Figure 2, upper panel taken the latter date; lower panel belongs to 1971). Boy! How we worked and sweated, learned and enjoyed it! Theoretical lectures, hands-on physiology and electronics lab exercises, evening study-discussion sessions ... LaNelle E. Nerger, then his soon wife to be and now his recent widow after 45 solar cycles of unbroken companionship, shared the experience from her nurse perspective that she was to later on enrich during an active professional life, which reached the doctoral level. The 6-week course had been designed by Les and by Dr. Hebbel E. Hoff (1907-1987), the latter then Chairman of the Department and another superb scientist, teacher and human being [2]. For years they were driving forces in outstanding consonance from who, anybody with motivation and enthusiasm, would climb up the scientific ladder. That course was offered without interruption during 17 years (1956-74) and was supported by NIH; approximately 700 professionals and a few students from several disciplines went through it. For many, it became a launching platform, even changing their lives' courses, including me [3].

**Figure 2:**
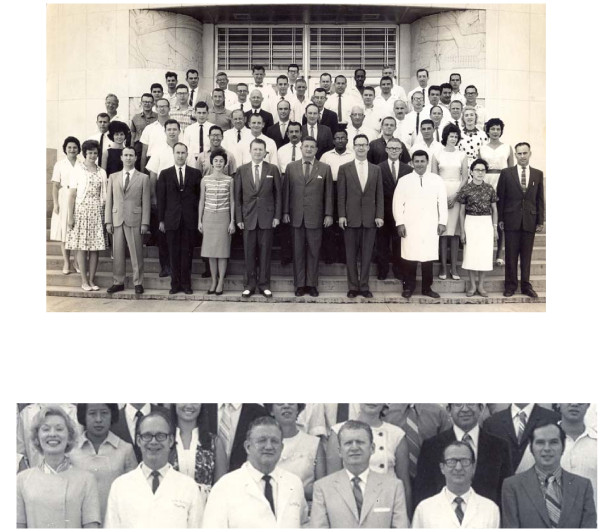
**The Summer Course in Houston**. Upper Panel: 1962 Class of the Summer Course. Picture taken at what used to be the main entrance of Baylor College of Medicine (BCM), in the Texas Medical Center (TMC). First row from left to right: Miss P. Olson (student), Max E. Valentinuzzi (student), Robert Wickham (secretary), Miss D. Greene, L.A. Geddes (Professor), Hebbel E. Hoff (Chairman), Lee E. Baker (Graduate Student), Ariel BarSela (MD), Beatriz Velázquez (Mexican student, MD), H. Hitchcock (student). First on the right hand, third row, LaNelle E. Nerger (student). Lower Panel: LaNelle E. Geddes, Dr. Lee E. Baker (Associate Professor), Dr. Hebbel E. Hoff (Chairman), Dr. Leslie A. Geddes (Professor), Max E. Valentinuzzi (Assistant Professor), Joe Bourland (Staff Member). During the 1971 Summer Course, same place as above.

The versatile Physiograph was the main equipment in those days, a fully electronic-mechanical three or four channel recorder [4] that could pick up a variety of physiological variables, such as ECG, EMG, blood pressure, cardiac contractions, smooth or skeletal muscle contractions, so permitting access to a wealth of physiological information. Soon the instrument spread all over the USA and abroad; besides, it was relatively inexpensive. It was accompanied by a didactic Laboratory Manual that beautifully assisted the students in their practical daily endeavors [5].

Les and his collaborators had introduced also the impedance pneumograph [6-8], which was a simplified and very practical version of previous developments made by others. It became a ubiquitous unit that trod many roads in the hands of eager and curious students leading to surprising and elegant results, as for example the Law of Impedance Pneumography [9] or the first on-line intraventricular pressure-volume diagrams [10,11] reminiscent of the typical loops encountered in thermodynamic machines. Such contributions were clear predecessors of the intracardiac diagrams, produced by the ingenious and successful conductance catheter later brought up by Jan Baan and colleagues, in Leiden, The Netherlands [12]. Swiftly come to my mind the well-trained dogs used on the treadmill by Carrie Palmer, Les' graduate student; *Peanuts*, a nice kind of female mixed German Shepherd, became a prominent character in the lab [13].

Ventricular fibrillation and especially its counterpart, defibrillation, stand out as subjects occupying his concern along the years. Many were the students recruited to such effort by Les' charismatic enthusiasm. Each year there was a field sojourn to the Veterinary School at College Station, TX, to run a magnificent whole day experiment on a horse (Figure 3). It was the middle of the Summer Course and, almost at the end of the day, a defibrillation attempt took place, each year increasing the delivered power and, disappointingly, each year the acting equine refusing to come back (remember, the procedure faced a big heart, in the order of 3 kg). The huge and always growing defibrillator became almost a legend. Long is the list of papers on the subject; at one point, an outstanding and still valid book, authored by Tacker and Geddes, in 1980, in many respects put together the collected information [14]. *Tack *Tacker, as we used to call him, one of his faithful collaborators during a lifetime together with Joe Bourland since the Baylor years. *Tack *is still at Purdue working with Charles Babbs, another in the team. Several other more recent contributions in this subject enriched clinical knowledge, one, for example, on a new cardioresuscitatory technique, also with a longstanding development [15-18]; in it, they investigated sustained abdominal compression as a means to improve coronary perfusion. A second one deals with degradation, indicated among other variables by a decrease in the fibrillation frequency, as ventricular fibrillation progresses [19].

**Figure 3:**
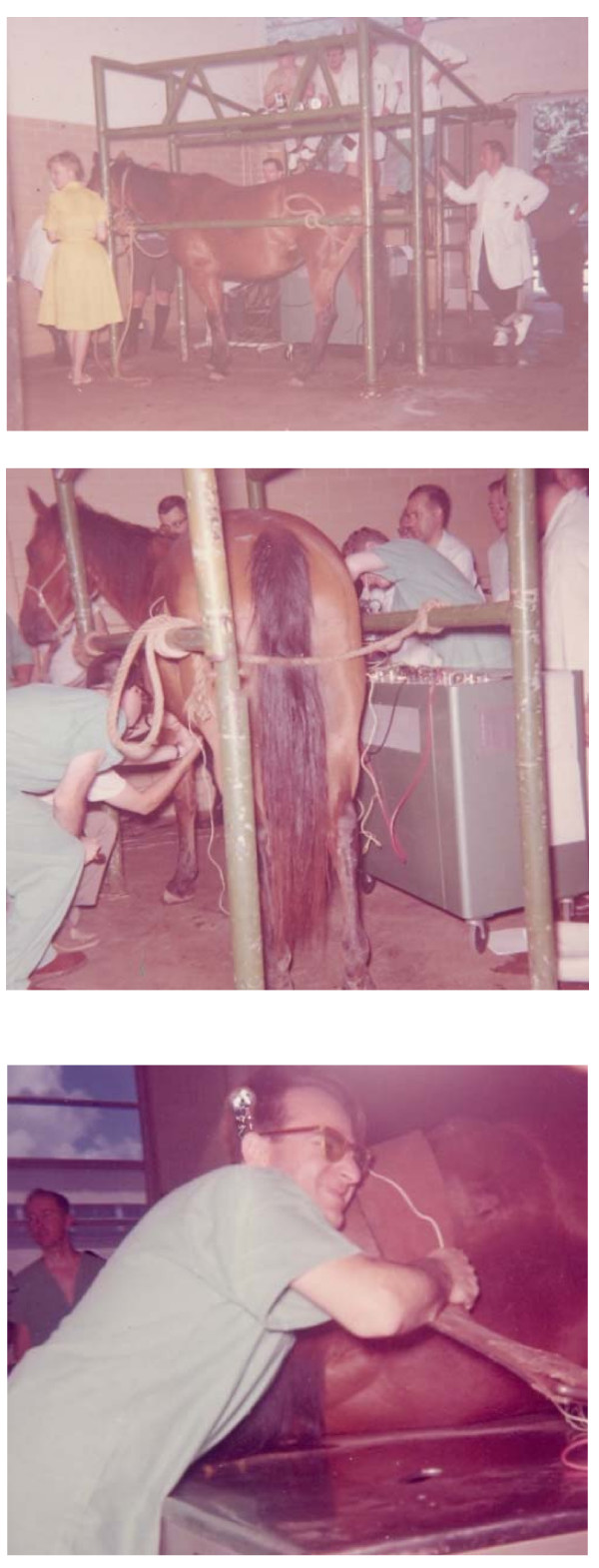
**1962 Class of the Summer Course**. Field trip to the Veterinary School at College Station, Texas A&M. Upper frame: Preparing the animal. LaNelle E. Nerger at the far left. Middle frame: Dr. Geddes probably attaching electrodes to the unanesthetized equine. Lower frame: Max Val performing the old Chauveau-Marey maneuver, that is, via the rectum, manually compressing the abdominal descending aorta to elicit heart slowing by vagal response. This maneuver led 10 years later to the Nightingale Prize mentioned in the text.

Physiological signals attracted considerable part of his activities because one of his perennial mottos was *measurement is essential in physiology*, followed right away by *first you must identify and unequivocally define the variable *(complemented with *that is why psychological stress is so difficult to quantify*), and *if you do not find a principle of transduction and an adequate technology for implementing it, the measurement results unviable*. Thus, the necessary and sufficient conditions for carrying out a given measurement were clearly stated, all this nicely and extensively presented in his books [20-22]. The properties of stimulating and recording electrodes had been a continuing interest since graduation from McGill, as for example using dry electrodes [23] or the peculiarities and wide spectrum offered by the electrocardiographic signal [24]. He has written thirteen books and over eight hundred scientific papers, receiving the Nightingale Prize in 1973 for one of them [25,26] and, thereafter, the Texas Medical Association Award (1974) for a videotape on acute myocardial infarction. Not only his interests stayed within the academic environment but an industrial hue was manifested in over 20 USA patents, all applied to medical use [27,28]. History of science and technology was another area in which, often with Hebbel Hoff, he uncovered astounding and delightful information, as for example papers on the graphic registration of physiological events [29-31] or his series of historical articles in the RETROSPECTROSCOPE Section of the *IEEE/EMB Magazine*, which in itself constitutes a solid set deserving to be collected within a special volume. I well and kindly remember, with a melancholy touch, the revival history experiments, as the arterial blood pressure measurement in the horse (and also in the dog) with the open manometer, as first done by Stephen Hales in 1728 along with the reading of Hales' first paragraph: *In December, I caused a mare tied down *... No, it is beyond my capability to review everything Les did, least of all what he did during the long span at Purdue; perhaps some well-trained biographer in the not too distant future will decide to tackle such a project making use of documents and archival material surely to be found at McGill, Baylor and Purdue Universities.

Still young, his family moved to Canada, where he earned a bachelor's degree in 1945 and a master's degree in 1953, both in electrical engineering, from McGill University in Montreal. Thereafter, in Houston as close collaborator of Hebbel E. Hoff, Les earned a doctorate in physiology in 1959 from Baylor University College of Medicine. Purdue University got hold of him for his last and longest period and made him Showalter Distinguished Professor of Biomedical Engineering. In 2006, Dr. Geddes was recipient of the National Medal of Technology; several other important and well deserved distinctions brilliantly covered his chest.

To underline and emphasize were the affection, kind devotion and concern for their difficulties shown to *His People*, even when mildly "spanking" someone for forgetfulness or for an eventual mishap; they were *his intellectual family*, undergraduates, graduate students, technicians of all levels, janitors, everybody rushed dutifully and happily after any of Les' requests, which always were softly said (Figure 4). Let me make these words a humble reminiscence to them, including also the many I never met at Purdue. Generosity appeared constantly. Take a look at the long list of publications and in many, very many, his name occupies a second or third or a fourth author position; authorship order was never a problem or a cause of friction in the old Department of Physiology and I am sure it was not at Purdue.

**Figure 4:**
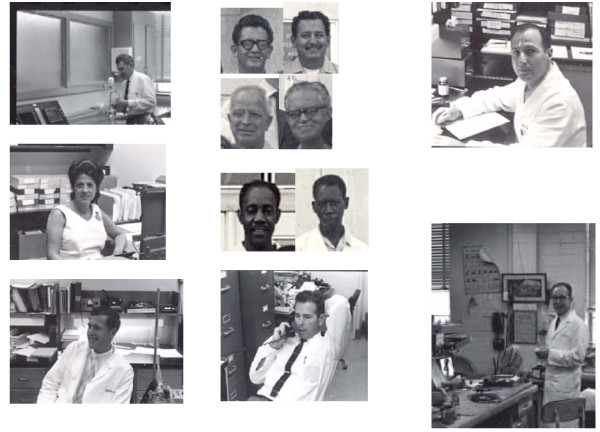
**Some of *His People *in the sixties, at Baylor**. Several names and photos are missing, as Garland Cantrell's, electronic technician. My apologies! Left column, top: Les in the student physiology lab. Center: Lucia Bonno, Les' secretary in the 60's. Bottom: Jeff Peters, Graduate Student in 1967-71, later became MD and worked with Dr. Kolff, in Utah. Middle column, top, clockwise: Ernesto Arriaga, Cruz Martínez, Arno Moore, John Vasku, Technicians. Center: Carter Jordan, left; Narvin Foster, right, Animal Technicians. Bottom: Tom Coulter, Electronic Technician. Right column, top: Max Valentinuzzi, Graduate Student, 1966-1969, Assistant Profesor, 1969-1973. Bottom: Dr. Lee Baker, Assistant Professor; later, Professor at the University of Texas, Austin.

The last time I talked with him over the phone (in September or October 2007 during one of my trips to the States), after asking him how he was, he answered cheerfully and full of pep ... *well, Max ... as everything in life, wearing out a little ... but not rusty! *Nice attitude, ain't it?

## Physiograph

by Rebecca Roeder

*One of Dr. Geddes' last graduate students at Purdue who started working with him in 1995. Written just days before he departed*; *reproduced with her kind permission*.

In Texas Geddes did arrive,

from Canada they made him drive

to work with the iron lung brigade; 

inventing equipment was his trade.

 

The kymograph needed replacing 

smoking drum for each tracing.  

Hebbel Hoff set forth the mission

Geddes' curiosity, intellect, intuition.

 

Differential amps, vacuum tubes

tucked in neatly packaged cubes.

High CMRR and immune from noise,

novel ideas it employs.

 

And thus, the Physiograph was created 

electrical and mechanical interrelated;

compatible with oscilloscope. 

Did you know he even helped the Pope?

 

Collecting data with great speed 

Ahh ... a lab manual is what we need.

Teaching physiology was the course,

wow ... the Chauveau-Marey in a horse!

 

Back to the big modular machine,

continuous chart or projecting screen,

 EEG, ECG, respiration, heart sounds, 

blood pressure at every station.

 

Turtle, camel, fly, snake

a graphic recording he did make.

Any subject he could find

there were data to be mined.

 

So, cover up your civilian dress,   

let's spray the unsuspecting guests,

with purple glory it will cover

anyone who dares to hover.

 

Teaching each biomedical principle,  

at the same time, complex and simple.

Stating the obvious is the key

A special mentor he was to me.
